# Reduction in LFP cross-frequency coupling between theta and gamma rhythms associated with impaired STP and LTP in a rat model of brain ischemia

**DOI:** 10.3389/fncom.2013.00027

**Published:** 2013-04-05

**Authors:** Xiaxia Xu, Chenguang Zheng, Tao Zhang

**Affiliations:** Computational Neuroscience Lab, The College of Life Sciences, Nankai UniversityTianjin, China

**Keywords:** two-vessel occlusion, cross frequency conditional mutual information (CF-CMI), synaptic plasticity, hippocampus, neural information flow (NIF)

## Abstract

The theta-gamma cross-frequency coupling (CFC) in hippocampus was reported to reflect memory process. In this study, we measured the CFC of hippocampal local field potentials (LFPs) in a two-vessel occlusion (2VO) rat model, combined with both amplitude and phase properties and associated with short and long-term plasticity indicating the memory function. Male Wistar rats were used and a 2VO model was established. STP and LTP were recorded in hippocampal CA3-CA1 pathway after LFPs were collected in both CA3 and CA1. Based on the data of relative power spectra and phase synchronization, it suggested that both the amplitude and phase coupling of either theta or gamma rhythm were involved in modulating the neural network in 2VO rats. In order to determine whether the CFC was also implicated in neural impairment in 2VO rats, the coupling of CA3 theta–CA1 gamma was measured by both phase-phase coupling (*n*:*m* phase synchronization) and phase-amplitude coupling. The attenuated CFC strength in 2VO rats implied the impaired neural communication in the coordination of theta-gamma entraining process. Moreover, compared with modulation index (MI) a novel algorithm named cross frequency conditional mutual information (CF-CMI), was developed to focus on the coupling between theta phase and the phase of gamma amplitude. The results suggest that the reduced CFC strength probably attributed to the disruption of the phase of CA1 gamma envelop. In conclusion, it implied that the phase coupling and CFC of hippocampal theta and gamma played an important role in supporting functions of neural network. Furthermore, synaptic plasticity on CA3-CA1 pathway was reduced in line with the decreased CFC strength from CA3 to CA1. It partly supported our hypothesis that directional CFC indicator might probably be used as a measure of synaptic plasticity.

## Introduction

Hippocampus is known to be one of the most important brain regions closely related to the learning and memory processes with synaptic plasticity as the accepted cellular basis (Howland and Wang, 2008; Shang et al., 2010; Sydow et al., 2011; Foster, 2012). One of the functional indices of synaptic plasticity is long term potentiation (LTP) (Quan et al., 2010), which is a long lasting enhancement of synaptic strength induced by high-frequency stimulating presynaptic neurons (Bliss and Lomo, 1973). In addition, the early transient potentiation phase of LTP lasting 10 min or less is termed short-term potentiation (STP) and is considered to be one candidate mechanism for short term memory (STM) (Erickson et al., 2010).

Synchronized neural oscillations were supposed to facilitate simultaneous firing of neural population and may be related to cognitive processes (Basar et al., 2001; Ward, 2003; Zhang, 2011). Conventionally, neural oscillation is classified into five frequency bands e.g., delta 1–4 Hz, theta 4–8 Hz, alpha 8–13 Hz, beta 13–30 Hz, and gamma 30–150 Hz (Buzsaki and Draguhn, 2004), which are possibly associated with different brain status. Among these rhythms, both theta and gamma rhythms in hippocampus, modulated during perception and memory tasks, are supposed to be most relevant to cognition (Kahana et al., 2001; Behrendt, 2010). We previously utilized an approach of general partial directed coherence (gPDC), which was one of directional algorithms, to determine the directionality of neural information flow (NIF) between CA3 and CA1 (Xu et al., 2012). It was found that coupling directional index was significantly reduced at either theta or gamma frequency bands between hippocampal CA3 and CA1 regions in brain ischemic rats, which might be associated with the alteration of LTP (Xu et al., 2012). In addition, a previous study showed that the coupling direction indices from thalamus to medial prefrontal cortex were considerably decreased at the theta rhythm in the rat model of depression, and increased after memantine treatment, which might be also associated with the LTP alterations and cognitive impairment (Zhang et al., 2011). However, so far the above NIF measurements of directional index have only been performed in a same frequency band rather than cross frequency bands. Accordingly, a question has been raised as to whether there is a causality relationship between rhythms, such as theta and gamma rhythms, between two brain regions.

Recently, several studies reported that there were two forms of cross frequency coupling (CFC) between theta and gamma rhythms, namely *n*:*m* phase-phase coupling (Belluscio et al., 2012) and phase-amplitude coupling(Canolty et al., 2006). It suggested that the alterations of CFC were possibly involved in the changes of cognitive function (Chrobak et al., 2000; Lisman, 2005; Sauseng et al., 2009). Modulation index approach (Canolty et al., 2006) can be employed to measure phase-amplitude coupling (PAC) between hippocampal CA3 and CA1. However, the measurement of modulation index is affected by both the amplitude and phase signals. Therefore, a novel measurement is needed, which focuses on the coupling between theta phase and the phase of gamma amplitude. In the present study, a novel approach, named cross frequency mutual information (CF-CMI), was developed based on conditional mutual information (Palus et al., 2001; Palus and Stefanovska, 2003). In contrast to an approach of MI, which transiently combines the amplitude envelope of high-frequency with the phase of low frequency rhythm into analytic signals, the approach of CF-CMI focuses on the phase–phase coupling between two different rhythms. This novel coupling measurement may provide an underlying indication of the coupling strength possibly corresponding to the information coding in hippocampus.

In this study, Male Wistar rats were used and the two vessel occlusion (2VO) (Xu et al., 2012) model was successfully established. Local field potentials were collected before STP and LTP performed on hippocampal CA3 and CA1 pathway. The phase locking value (PLV) measurement was used to measure the phase synchronization between CA3 and CA1 regions over a particular rhythm, such as theta or gamma rhythm. In order to determine whether the CFC was also implicated in neural impairment in 2VO rats, we examined the theta-gamma coupling between CA3 and CA1 in hippocampus, which were done by both phase-phase coupling (*n*:*m* phase synchronization) and PAC. Furthermore, the CF-CMI was used to measure the coupling strength between theta phase and the phase of gamma amplitude. An issue was addressed as to whether such a directional index of NIF between cross-frequency bands is able to reveal the variations of hippocampal synaptic plasticity in brain ischemia, combining with the alterations of STP and LTP on CA3-to-CA1 neural pathway.

## Materials and methods

### Experimental animals

Experiments were performed on male Wistar rats (280–300 g, around 8-week old), which were provided from the Laboratory Animal Center; Academy of Military Medical Science of People's Liberation Army, and reared in the animal house of Medical School, Nankai University. Animals were housed in a 12 h light/dark cycle with freely feed and water and randomly divided into two groups (*n* = 12), namely Con group (*n* = 6) and 2VO group (*n* = 6). A rat model of 2VO was established, which was as same that in our previous reports (Li et al., 2011; Xu et al., 2012). Rats were reared for 3 weeks since operation. All procedures were carried out in accordance with the Ethical Commission at Nankai University, China.

### Electrophysiological experiment

Rats was placed in a stereotaxic frame (Narishige, Japan) under 30% urethane anesthesia (4 ml/kg, i.p., Sigma-Aldrich, St. Louis, MO, USA). The skull was opened and a small hole (2 mm in diameter) in its left side was drilled. Two Stainless steel electrodes were slowly implanted into CA3 and CA1 sites (CA3: 4.2 mm posterior to the bregma, 3.5 mm lateral to midline, 2.5 mm ventral below the dura; CA1: 3.5 mm posterior to the bregma, 2.5 mm lateral to midline, 2.0 mm ventral below the dura), respectively. Ground and reference electrodes were placed symmetrically over the two hemispheres of the cerebellum. The signals of local field potential were collected concurrently from the regions of CA3 and CA1 at a sampling rate of 1000 Hz.

After LFPs were collected, STP and LTP recordings were performed in the same brain regions. First, low-frequency stimulations (0.05 Hz) for 20 min were delivered to Schaffer collateral evoking a response of 50% of its maximum. And then tetanic stimulation (10 pulses at 100 Hz for 2 s repeated 10 times) was delivered and field excitatory postsynaptic potentials (fEPSPs) were recorded at 20 kHz sampling rate every 20 s for 60 min. fEPSPs slope was used to measure synaptic efficacy (Li et al., 2011). As the average responses, STP and LTP were measured at the first 10 min and between 50 min and 60 min after induction, respectively. The initial data was analyzed by Clampfit 9.0 (Molecular Devices, Sunnyvale, CA, USA).

### Phase locking value (PLV)

PLV is a widely used method to measure the strength of phase synchronization within rhythms between brain regions (Rosenblum et al., 1996). φ_*a*_ and φ_*b*_ signed the phase of the two signals and PLV is defined as

$$PLV=\left|\frac{1}{N}\sum_{j=1}^{N}\exp(i[\phi_{a}(j\Delta t)-\phi_{b}(j\Delta t)])\right|$$

*N* stands for the length of the signal and $\frac{1}{\Delta t}$ is the sampling frequency. The value of PLV is within [0, 1] with 1 indicates fully synch and 0 no syncing at all.

### *n:m* phase synchronization

Cross frequency phase-phase coupling between theta and gamma rhythms was determined by *n*:*m* phase synchronization, where the ration of *n*:*m* stood for stable *n* cycles of the gamma oscillator for every *m* theta oscillator.

The radial distance (*r*) values, determined as: $r_{n:m}=\left|\frac{1}{N}\sum_{t=1}^{N}e^{i\left[m\;\times \;\phi_{\text{theta}}(t)\;-\;n\;\times \;\phi_{\text{gamma}}(t)\right]}\right|$ were used to determine the strength of cross frequency phase-phase coupling.

The distribution of *r*_*n:m*_ for different rations, e.g., 1:1, 1:2, …, 1:10, etc. was calculated. A Larger value of *r* indicated a more unimodal distribution of Δϕ_*n:m*_(*t*) = *m* × φ_theta_(*t*) − *n* × φ_gamma_(*t*), i.e., stronger phase coupling (Rayleigh test for uniformity) (Tass et al., 1998; Belluscio et al., 2012).

### Phase amplitude coupling (PAC)

Modulation index (MI) was used to evaluate the cross frequency PAC between CA3 and CA1 regions. The main idea of MI measure was to create a composite signal with amplitude envelope of the high frequency (*A*_famp_(*t*)) as its amplitude and instantaneous phase of the low frequency (φ_fph_(*t*)) as its phase.

$$Z_{\text{fph},\text{ famp}}(t)=A_{\text{famp}}(t)\times \exp(i\times \phi_{\text{fph}}(t))$$

This composite signal created a joint probability density function on the complex plane. The initial value of MI is calculated as the absolute value of the average of the composite signal:

$$MI_{\text{raw}}=abs\;(\text{mean}(Z_{\text{fph},\text{ fam}}(t)))$$

For further processing, surrogate data need to be generated by bringing a random time lag τ between φ_fph_(*t*) and *A*_famp_(*t*): *Z*_surr_(*t*, τ) = *A*_famp_(*t* + τ) × exp(*i* × φ_fph_(*t*)).

Finally, MI is defined as *MI* = (*MI*_raw_ − μ)/σ, where μ is the mean of the surrogate lengths and σ is a standard deviation.

In this case, Morlet wavelets of the depth 7 were applied to generate analytic representations with a frequency range of 1–20 Hz in CA3 and 30–80 Hz in CA1. And then Hilbert transform was used to obtain CA3 φ_fph_(*t*) and CA1 *A*_famp_(*t*), respectively. Finally, a window length of 40 s with 50% overlap and 100 trials of surrogate data were employed in the study.

### Phase-amplitude coupling based on conditional mutual information

In order to measure the strength of directional CFC between CA3 and CA1 regions, an improved algorithm named cross frequency conditional mutual information (CF-CMI) was made. Specifically, we firstly extracted the phase of broadband-filtered theta rhythm (from 4 Hz to 8 Hz) in CA3 region (φ_theta_) and the amplitude of the narrowband-filtered gamma rhythm (from 30 Hz to 80 Hz, step = 1 Hz) in CA1 region (amp_gamma_) by Hilbert transformation. Since amp_gamma_ did not vary very fast, we band-filtered it from 1 Hz to 10 Hz. And then the phase of amp_gamma_ was extracted by a second Hilbert transformation signed as φ_amp_γ__. Finally, CMI (Palus et al., 2001; Palus and Stefanovska, 2003) was applied to measure the directional coupling between φ_theta_ and φ_amp_γ__.

Briefly, supposing two processes {*X*_*CA*3_} and {*X*_*CA*1_} (from the amplitude envelope of signals in CA1), their instantaneous phases {ϕ_theta_} and {φ_amp_*r*__} can be estimated by application of the discrete Hilbert transform. Accordingly, the “net” information about the τ − future of the process {φ_amp_*r*__} contained in process {φ_theta_} using *C* = *I*(φ_theta_; Δ_τ_φ_amp_γ__|φ_amp_γ__).

To establish possible causality relations, we consider phase increments,

$$\Delta_{\tau }\phi_{{\text{amp}}_{\gamma }}=|\phi_{{\text{amp}}_{\gamma }}(t+\tau )-\phi_{{\text{amp}}_{\gamma }}(t)$$

Then the conditional mutual information is defined as, *I*(φ_theta_; Δ_τ_φ_amp_γ__|φ_amp_γ__) = *H*(φ_theta_|φ_amp_γ__) + *H*(Δ_τ_φ_amp_γ__|φ_amp_γ__) − *H*(φ_theta_, Δ_τ_φ_amp_γ__|φ_amp_γ__).

### Data and statistical analysis

All data were presented as mean ± SEM. Of the STP and LTP test, field excitatory postsynaptic potentials (fEPSPs) slopes were expressed as the percentage change of the baseline. Statistical comparisons were made using the Wilcoxon rank sum test. The analyses were performed using SPSS 17.0 software with the significant level setting at *P* < 0.05.

## Results

**Figure 1:**
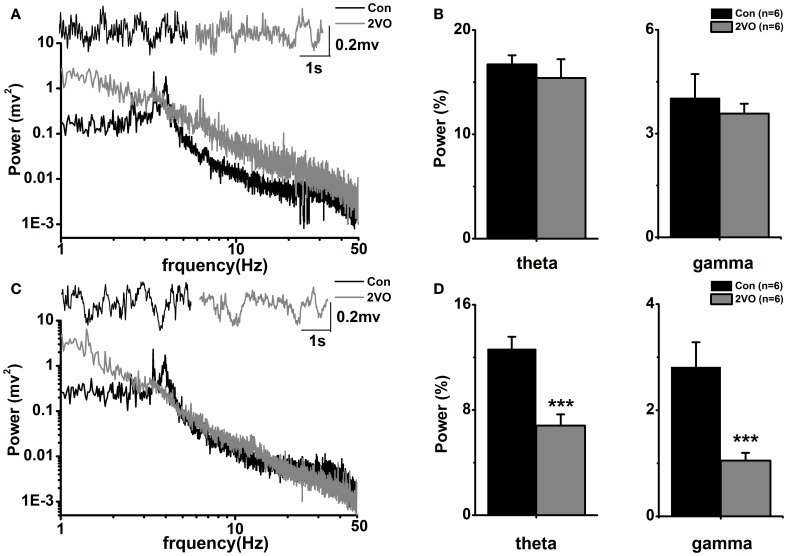
**Power spectral analysis in the two groups. (A)** Representative local field potential traces and corresponding power spectra in hippocampal CA1 regions in one normal rat (black line) and one 2VO rat (gray line). **(B)** Statistical analysis of relative theta and gamma power spectra in CA1 region in the two groups. **(C)** Same display as **(A)** in CA3 region. **(D)** Same display as **(B)** in CA3 region. ^***^p < 0.001 comparison between Con and 2VO groups.

Traces show representative sections of original neurograms obtained from recordings of LFPs made one normal Wistar rat at hippocampal CA1 region (black line in upper panel of Figure 1A) and CA3 area (black line in upper panel of Figure 1C) as well as a 2VO rat at CA1 (gray line in upper panel of Figure 1A) and CA3 (gray line in upper panel of Figure 1C). The signals were obtained at 1000 Hz sampling frequency and a 5 s sampling period.

### Power spectrum of LFP

Digitized LFPs signals were subjected off-line to a fast Fourier transformation to produce a power spectrum. Based on Wilcoxon rank sum test, it shows that there is no significant difference of total power between Con group and 2VO group in either theta frequency band (4–8 Hz) or slow gamma frequency band (30–50 Hz) in CA1 region (Figure 1B). In addition, there are significant decreases of total power in both theta and slow gamma frequency bands in 2VO group compared to that in Con group in hippocampal CA3 region (theta, *F* = −2.882, *p* = 0.004; gamma, *F* = −2.882, *p* = 0.004, Figure 1D).

### Phase synchronization

**Figure 2:**
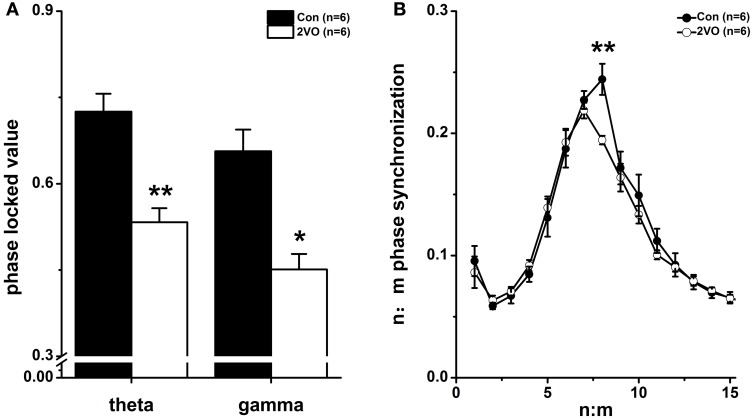
**Phase synchronization index. (A)** Phase locking value (PLV) of LFPs between CA3 and CA1 at theta and gamma frequency bands in Con and 2VO groups (*n* = 6). **(B)** Phase–phase (*n*:*m*) coupling between theta and gamma oscillations. Mean radial distance values (*r* values) from the distribution of the difference between *m* × theta and *n* × gamma phases calculated for different ratios in these two groups. ^*^p < 0.05 and ^**^p < 0.01 comparison between Con group and 2VO group.

Figure 2A showed the phase synchronization analysis at theta and slow gamma frequency bands for control and 2VO groups. The original signals were filtered into 1–50 Hz range (bandwidth = 1 Hz, step = 1 Hz). Based on the Hilbert transform, the phases of the filtered signals were generated and then used to compute the PLV. It was found that PLVs at both theta and gamma frequency bands were much lower in 2VO group compared to that in Con group (theta: *F* = −2.882, *p* = 0.004; gamma: *F* = −2.562, *p* = 0.010, Figure 2A).

### Cross frequency phase–phase coupling

With the purpose of investigating the cross frequency theta-gamma phase coupling quantitatively, the radial distance values (*r*) of the circular distribution from the phase differences between *m* × theta (CA3) and *n* × low gamma (CA1) phases for 15 ratios were calculated (Figure 2B). Rayleigh test showed that there were a distinct peak at *n:m* = 1:8 ratio (*p* < 0.05) in Con group and another peak at *n:m* = 1.7 (*p* < 0.05) in 2VO group. Furthermore, Wilcoxon rank sum test showed that there was a significant difference of 1:8 phase synchronization values between these two groups (*F* = −2.882, *p* = 0.004). It implied that cross frequency phase–phase coupling might be weakened in brain ischemia rats.

### Cross frequency phase-amplitude coupling

**Figure 3:**
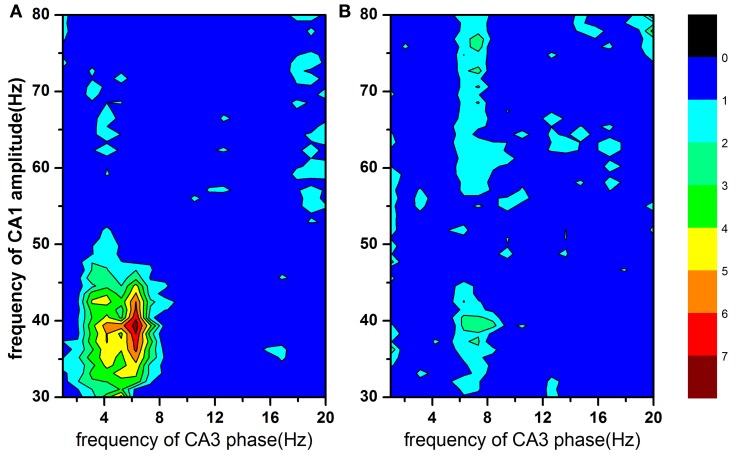
**The modulation index as a function of analytic amplitude (30–80 Hz) in CA1 and analytic phase (1–20 Hz) in CA3.** Larger MI value indicates stronger cross frequency coupling. Strong phase-amplitude coupling between CA3 and CA1 existed at CA1 slow gamma band (30–50 Hz) in normal rats (panel **A**), however, almost disappeared in brain ischemia rats (panel **B**).

Figure 3 showed the mean modulation indices in both Con and 2VO groups, which reflected cross frequency PAC between CA3 phase sequences (1–20 Hz, step = 1 Hz) and CA1 amplitude sequences (30–80 Hz, step = 1 Hz). Larger values of MI indicate stronger cross frequency coupling. In normal animals, the maximal coupling was found at both 40 Hz of CA1 amplitude and 6 Hz of CA3 phase (Figure 3A), while the strong PAC between CA3 and CA1 existed at slow gamma band (30–50Hz). However, this cross frequency PAC was almost disappeared in brain ischemic rats (Figure 3B).

### Reduced phase-amplitude directional coupling associated with impaired STP and LTP

**Figure 4:**
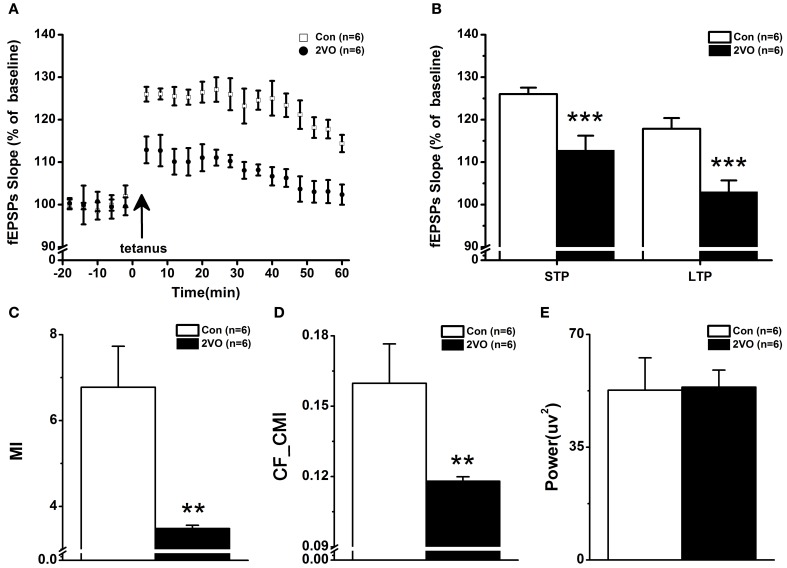
**The impaired synaptic plasticity in CA3-CA1 pathway paralleling with the decreased directional CFC index between CA3 theta rhythm and CA1 gamma rhythm in 2VO group. (A)** STP and LTP were elicited by the tetanic stimulation indicated by the arrow. The fEPSPs slope was normalized to baseline. **(B)** Magnitudes of STP and LTP, determined as responses between 0 and 10 min and between 50 and 60 min after tetanic stimulation, were significantly smaller in 2VO rats. **(C)** CFC analysis measured by MI method between CA3 theta and CA1 gamma rhythms in the two groups. Considerably decreased MI values could be seen in 2VO group. **(D)** Statistical results of CA3 gamma power spectra in one CA1 theta cycle between the two groups. A difference that was no statistically significant could be seen. **(E)** Directional CFC index from CA3 theta rhythm to CA1 gamma rhythm measured by CF-CMI was significantly reduced in 2VO rats. ^**^p < 0.01 and ^***^p < 0.001 comparison between Con and 2VO groups.

Stimulating Schaffer collateral evokes basal field excitatory postsynaptic potentials (fEPSPs) in the hippocampal CA1 region. Figure 4A shows the time courses of fEPSPs slopes normalized to the 20 min baseline period. It can be seen that the fEPSPs slopes are increased immediately after the high-frequency stimulation and then stabilized to a level above the baseline period. The mean fEPSP slopes of the first 10 min after HFS were examined as STP results. Based on Wilcoxon rank sum test, it was found that the mean fEPSPs slope was lower in 2VO group than that in control group (113 ± 3.42% vs. 126 ± 1.51%, *p* < 0.001, Figure 4B-left). Furthermore, LTP was measured as the mean fEPSP slopes in 45–60 min after HFS. It could be seen that the mean fEPSPs slope was much lower in 2VO group than that in control group (103 ± 2.65% vs. 118 ± 0.50%, *p* < 0.001, Figure 4B-right).

Figures 4C–E shows the data of statistical CFC analysis. It was found that the value of MI was enormously lower in 2VO group compared to that in control one (*F* = −2.882, *p* = 0.004, Figure 4C). In order to measure the directional cross-frequency coupling (CFC) between theta rhythm in CA3 and gamma rhythm in CA1, LFP signals were filtered over 1–50 Hz with 1 Hz bandwidth, using FIR band filter with hamming window (filter order = 512). Two types of phase sequence were extracted by means of Hilbert transform, one from original LFP signals within theta frequency band and another from the amplitude of LFP signals within gamma frequency band. And the novel algorithm of CF-CMI was applied to determine the directionality of NIL between these two areas. It can be seen that the value of CF-CMI measurement is much lower in 2VO rats compared to that in control animals (*F* = −2.882, *p* = 0.004, Figure 4D). There was no statistical difference of gamma power spectra in one theta circle between these two groups (Figure 4D).

## Discussion

In this study, a 2VO rat model was employed with impairments cognition functions (Li et al., 2011). In addition, a novel algorithm was developed to measure the CFC directionality between CA3 and CA1 regions in hippocampus. It was found that the CFC directional index from CA3 theta rhythm to CA1 gamma rhythm was significantly reduced, which was interestingly in line with the alteration of STP and LTP in CA3-CA1 pathway in brain ischemic state. The above result shows great promise for our hypothesis that the CFC directionality could be an indicator of the synaptic plasticity in hippocampal CA3-CA1 pathway.

Phase synchronization within both theta and gamma rhythms was believed to be crucial to the cognitive behaviors (Basar-Eroglu et al., 1992; Gallinat et al., 2006), while cognitive impairment usually accompanied with reduced phase synchronization (Yener et al., 2007; Ford et al., 2008). In the present study, it was found that both theta and gamma synchronizations were considerably decreased in 2VO group compared to that in Con group (Figure 2A), implying that there was a disturbance of neural synchronized coordination in brain ischemic state. The fact that the reduction of phase synchronization was associated with cognitive deficits was in line with the findings in Schizophrenia and Alzheimer subjects (Yener et al., 2007; Ford et al., 2008). Moreover, the analysis of cross frequency phase coupling (Belluscio et al., 2012) showed that the *n:m* (1:8) theta-gamma rhythm coding in Con group was changed to the *n:m* (1:7) in 2VO group (Figure 2B). Previous studies indicated that in computational models, identical gamma cycles with an equal number of spikes in each cycle were distributed across the entire theta cycle to support a multi-item working memory buffer (Lisman and Idiart, 1995; Jensen and Lisman, 1996). Each gamma cycle contains a discrete item (or position in space), and approximately seven gamma cycles could store 7 ± 2 sequential items. Thus, the reduction of the ratio might imply the impairment of memory capacity. However, the underlying physiological mechanism is still under further investigation. Our result of reduced ratios between theta and gamma rhythms (from 8:1 to 7:1, Figure 2B) in 2VO rats might indicate the impaired memory capacity (Sauseng et al., 2009) induced by 2VO operation.

Another form of CFC is the amplitude of gamma rhythm nesting in theta cycles, measured by modulation index (Bragin et al., 1995; Lakatos et al., 2005; Mormann et al., 2005; Canolty et al., 2006). One speculation of this coupling was that because of relative long conduction delays, theta rhythm was well suited to synchronize the networks over long distances while gamma rhythm nested in the theta cycle to coordinate cell assemblies involved in information dissemination process (von Stein and Sarnthein, 2000). In this study, it was found that CA1 low gamma rhythm, however not the high gamma rhythm, significantly nested in CA3 theta rhythm in Con rats (Figure 3A). Theta-gamma coupling was supposed to be relevant to cognitive function (Palva et al., 2005, 2010; Sauseng et al., 2008). In addition, it was reported that the low gamma rhythm was coherent between CA3 and CA1 in hippocampus, entrained by theta phase (Colgin et al., 2009). Therefore, we focused on the alteration of theta-gamma coupling in CA3-CA1 pathway associated with the cognitive disorder by 2VO. interestingly, such coupling phenomenon disappeared in brain ischemic state (Figure 3B), suggesting that the impaired cognitive function in 2VO rats was relevant to decreased theta-gamma coupling in CA3-CA1 pathway. Meanwhile, we did not pay attention to other neural pathways, such as cortico-hippocampal interactions and/or hippocampal DG-CA3 interactions, at the present study.

It is well known that there is a directional alteration of neural information flow, so as to measure directional CFC is more important to explore the relationship between the patterns of neural oscillation and cognitive functions. In our previous study, the algorithm of general partial directed coherence was utilized to determine the directionality of NIF between hippocampal CA3 and CA1 over either theta or gamma frequency band (Xu et al., 2012). We found that the coupling directional index was considerably decreased in the above two frequency bands in brain ischemic state, respectively. It was indicating that the strength of CA3 driving CA1 was significantly reduced. Subsequently, a hypothesis was raised that there was causality relationship in cross-frequency between hippocampal CA3 and CA1. MI algorithm has been used to measure CFC. From its formula, it can be seen that there are two factors affecting MI measurement. One is the cross phase coupling between these two frequency bands, and another is the amplitude of the high frequency band. Obviously, it will be better if we can distinguish between these two factors during the measurement of CFC.

In the present study, a novel algorithm CF-CMI is focused on measuring the coupling between theta phase and phase of gamma amplitude. Given that conditional mutual information is a directional algorithm over an identical frequency band, the developed CF-CMI should be a unidirectional coupling measurement across different frequency bands between two brain regions. Our data showed that there were no significant differences of the gamma power spectra in one theta circle between the two groups (Figure 4E). However, CF-CMI measurement presented that the value of directional CFC was much lower in 2VO group than that in Con group (Figure 4D), indicating that it was the phase information of signals rather than the amplitude of signal, which played an essential role in changing STP and LTP on CA3-CA1 pathway in brain ischemic rats (Figure 4B). The data further implied that the decreased information transmission along the CA3-CA1 pathway in cross-frequency of theta and slow gamma rhythms might be related to the impairment of STP and LTP in 2VO rats.

Taken together, our findings suggest that cognitive deficits caused by brain ischemia, such as learning and memory dysfunction, are implicated in the alteration of phase-phase coupling strength in theta and gamma oscillations. Moreover, the CA3-CA1 synaptic plasticity is impaired, which is in line with the decreased directional CFC from CA3 theta rhythm to CA1 gamma rhythm. It suggests that the modifications of diverse brain rhythms and their interaction, such as theta and gamma, are involved in regulating the behavioral functions. In addition, combining the impaired synaptic plasticity and reduced values of directional CFC, we would be able to understand that the directional CFC is likely to be another indicator of synaptic plasticity compared to that of NIF directionality obtained from same oscillatory rhythm. However, studying the relationship between the directional CFC and synaptic plasticity is still at an early stage of development. It remains open issues as to if there are other brain rhythms involved, which may indicate an alteration of cognitive functions.

### Conflict of interest statement

The authors declare that the research was conducted in the absence of any commercial or financial relationships that could be construed as a potential conflict of interest.

## Abbreviations

CFC: cross frequency coupling
CF-CMI: cross frequency conditional mutual information
fEPSP: field excitatory postsynaptic potential
CMI: conditional mutual information
LFP: local field potential
LTP: long term potentiation
MWM: Morris water maze
MI: modulation index
NIF: neural information flow
PAC: phase-amplitude coupling
PLV: phase locking value
STM: short term memory
STP: short term potentiation
2VO: two vessel occlusion.
